# THE EFFECT OF SOCIAL PARTICIPATION OF OLDER ADULTS ON DEPRESSION: FOCUSED ON THE MODERATING EFFECT OF HOUSEHOLD TYPE

**DOI:** 10.1093/geroni/igae098.3899

**Published:** 2024-12-31

**Authors:** Sehyun Baek, Sungkyu Lee

**Affiliations:** New York University, New York, New York, United States; Soongsil University, Seoul, Republic of Korea

## Abstract

Previous studies have found that the social participation of older adults is positively associated with perceived health, happiness, and life satisfaction (Kim et al., 2018). However, as they age, their social activities tend to decrease, leading to increased depression (Choi & Kim, 2023; Kim & Jung, 2017). Given that older adults living alone show a gradual decline in social activities as they age (Shin, 2022), the household type may intervene in the relationship between social participation and depression among older adults. However, little is known about the structural associations of these variables. Therefore, we examined the extent to which household type (i.e., living alone vs. not living alone) moderates the relationship between social participation and depression among older adults. We utilized data from the 2022 Korean Longitudinal Study of Aging (n=6,055) and conducted moderated regression analysis using Process Macro. The study found a negative relationship between social participation and depression of older adults (b=-.136, p<.01), and a moderating effect of household types on this relationship was statistically significant (p <.05). More specifically, the effect of social participation on depression was greater for older adults living alone than for those who do not live alone. These results highlight the need for policy efforts aimed at increasing the social participation of older adults to reduce their depression. Also, considering that the negative association between social participation and depression is more pronounced among older adults living alone, the interventions targeting the growing number of older adults living alone should be prioritized in policy planning.

